# Tick-borne encephalitis in Norway: A cohort study of clinical course and health-related quality of life at three- and twelve-month follow-up

**DOI:** 10.1007/s10096-025-05341-z

**Published:** 2025-11-08

**Authors:** Hilde Skudal, Tore Stenstad, Åslaug Rudjord Lorentzen, Else Quist-Paulsen, Jens Egeland, Børre Fevang, Keson Jaioun, Malin Veje, Bjørn Åsheim Hansen, Anne Marit Solheim, Hege Kersten, Randi Eikeland

**Affiliations:** 1 Department of Infectious Diseases, Telemark Hospital Trust, Skien, Norway; 2 Faculty of Medicine, University of Oslo, Institute of Clinical Medicine, Oslo, Norway; 3 Department of Infectious Diseases, Vestfold Hospital Trust, Tønsberg, Norway; 4 Sørlandet Hospital Trust, Norwegian National Advisory Unit on Tick-borne Diseases, Kristiansand, Norway; 5 Department of Neurology, Sørlandet Hospital Trust, Kristiansand, Norway; 6 Department of Microbiology, Oslo University Hospital, Oslo, Norway; 7 Division of Mental Health and Addiction, Vestfold Hospital Trust, Tønsberg, Norway; 8 Department of Psychology, University of Oslo, Oslo, Norway; 9 Department of Rheumatology, Dermatology and Infectious Diseases, Section of Clinical Immunology and Infectious Diseases, Oslo University Hospital, Oslo, Norway; 10 Department of Research, Telemark Hospital Trust, Skien, Norway; 11 Department of Infectious Diseases, Sahlgrenska University Hospital, Region Västra Götaland, Gothenburg, Sweden; 12 Department of Infectious Diseases, University of Gothenburg, Institute of Biomedicine, Gothenburg, Sweden; 13 University of Bergen, Institute of Clinical Medicine, Bergen, Norway; 14 Faculty of Health and Sport Sciences, University of Agder, Grimstad, Norway

**Keywords:** Tick-borne encephalitis, TBE, Neuroinfection, Long-term outcome, Quality of life

## Abstract

**Purpose:**

Knowledge of tick-borne encephalitis (TBE) prognosis is limited. This study aimed to describe the disease course in the first year and assess one-year outcomes, focusing on clinical recovery and health-related quality of life (HR-QoL).

**Methods:**

In this cohort study, we recruited hospitalized patients ≥ 16 years with confirmed TBE from hospitals in Norway’s endemic area. A composite clinical score consisting of variables on symptoms and neurological findings was scored at baseline (during hospitalization), 3- and 12 months. HR-QoL at 12 months was measured using RAND 36-item short form health survey and compared to the Norwegian reference population.

**Results:**

Among the 93 patients included, clinical improvement from baseline to 3 months was 68%, increasing to 77% by 12 months. The proportion of patients with symptoms or neurological findings influencing daily life was 98% at baseline, 52% at 3 months, and 41% at 12 months. 14% required inpatient rehabilitation, and 4/56 (7%) of active workers were on full-time sick leave at 12 months. Severe disease and comorbidities were linked to poorer outcomes. Most common symptoms influencing daily life at 12 months were fatigue (28%), concentration issues (13%) memory and sleep difficulties (12% each), while 8% had clinical findings where impaired balance and tremor dominated. Patients scored lower in physical health and social functioning regarding HR-QoL than reference population.

**Conclusion:**

Most improvements occur during the first three months; however, 41% of patients experience ongoing complaints at 12 months, impacting HR-QoL regarding physical health. Severe disease and comorbidities correlate with poorer prognoses.

**Trial registration:**

Project #2,296,959 **– **The NOrwegian Tick-borne Encephalitis Study – NOTES. An Observational Study on Clinical Features, Long-term Outcomes and Immune Characteristics – Cristin.

**Supplementary Information:**

The online version contains supplementary material available at 10.1007/s10096-025-05341-z.

## Introduction

Tick-borne encephalitis (TBE) is the most important arboviral disease in Europe [1], known for leading to severe central nervous system (CNS) infections. It is caused by the tick-borne encephalitis virus (TBEV). Transmission to humans occurs mainly from tick-bites and occasionally by consumption of unpasteurized dairy products [1, 2]. TBE often presents as a biphasic course, starting with a febrile illness with fever, headache, and myalgia, followed by an asymptomatic period lasting up to one week [2, 3]. Recent publications indicate that CNS involvement develops in the majority of patients with symptomatic TBEV infection [4, 5], presenting as meningitis, encephalitis, myelitis, or a combination thereof.

Three main subtypes of TBEV, named for their primary distribution areas, are European, Siberian, and Far Eastern [1]. Although infections from all subtypes can lead to both mild and severe TBE, the European subtype generally causes the mildest form, with a mortality rate of less than 1% [2]. In contrast, the Siberian subtype can cause chronic viral infection and the Far Eastern is considered to cause the most severe course with a case fatality rate up to 40% [1].

Despite the availability of an effective vaccine, TBE cases in Europe have markedly increased over the last decade, reaching 3,690 registered cases in 2023 [6]. Studies have shown that sequelae following TBE affect 40–50% of patients [7–9]. In Norway, TBE was rare until 2018, but cases have increased, peaking at 122 in 2023 [10]. In endemic regions of Norway, TBEV is currently the most common viral cause of CNS infection among adults with a molecularly or serologically confirmed diagnosis (unpublished data from Telemark, Vestfold and Sørlandet Hospital).

In 2020, the project “The Norwegian Tick-borne Encephalitis Study” (NOTES) was established to study TBE in Norway. The NOTES project group includes members from Vestfold, Sørlandet, and Telemark hospitals, covering all endemic regions in Norway. In 2024, NOTES published the description of the acute clinical characteristics of TBE [11], based on a population that accounted for 81% of adult hospitalized TBE patients in Norway over a five-year period. In the present study, we describe short- and long-term outcomes from this cohort to better understand the natural course of TBE during the first year after acute disease. Additionally, we assess patients’ experiences related to health-related quality of life and fatigue post-TBE. Currently, there is no standardized follow-up or framework to best describe and manage sequelae after TBE. The results of this study can contribute to establishing clinical guidance, assist in informing patients about their prognosis, raise awareness of vaccination and support the development of tailored rehabilitation programs.

## Materials and methods

### Setting and participants

This multicenter cohort study followed TBE patients from the acute phase during hospitalization and at 3- and 12-month follow-up after admission. Patients hospitalized to one of the three hospitals providing acute care in the region endemic for TBE —Sørlandet -, Vestfold -, and Telemark Hospital —during the period from January 2018 to December 2022 were asked to participate in the study. A retrospective cohort was collected between 2018 and 2020, but only patients who were included prospectively were asked to participate in this follow-up study.

The inclusion criteria were: (i) confirmed TBE according to the ECDC case definition [12]; clinical signs of CNS infection (e.g., meningitis, meningoencephalitis, meningoencephalomyelitis, encephaloradiculitis, meningomyelitis) and at least one of the following laboratory criteria; TBEV-IgM and TBEV-IgG antibodies in blood and/or TBEV-IgM in the cerebrospinal fluid (CSF) and/or seroconversion of TBEV-IgG in paired serum samples or detection of TBEV RNA by real-time polymerase chain reaction (PCR) in clinical specimens; (ii) aged ≥ 16 years; (iii) verified or suspected exposure to ticks in southeastern Norway; and (iv) hospitalized in participating hospitals. Patients vaccinated for TBE were included if infection with TBEV was confirmed by either intrathecal production of TBEV-IgG or TBEV-IgM in CSF or detection of TBEV-IgM in CSF or ≥ 10 months since the last vaccination dose was given when detection of both TBEV-IgG and TBEV-IgM in serum [13], along with clinical and laboratory findings (CSF pleocytosis >5 × 10^6^ cells/L) supporting the diagnosis.

### Disease classification

In the acute phase the patients were classified into three groups according to severity; mild disease was defined as meningeal symptoms with fever, headache, nausea, vomiting, neck stiffness, and light/sound sensitivity, with either normal or not performed electroencephalography (EEG). Moderate disease was defined as slightly altered consciousness or focal neurological symptoms. Severe disease was defined as multifocal symptoms and/or severe signs of encephalitis with altered consciousness as previously published [11]. Cognitive symptoms were assessed through clinical observation and categorized into mental slowness, altered consciousness and confusion. Symptoms and/or findings consistent with myelitis were evaluated clinically.

### Data collection

Four specialists in neurology and infectious diseases at the participating centers identified and investigated eligible patients. Only data from patients who attended both the 3- and 12-month visits were included in further analyses. Data was obtained by structural interviews, validated questionnaires and clinical investigations during the hospital stay for acute TBE and at 3- and 12-month follow-up visits. A modified composite clinical score (CCS) originally developed for assessing neuroborreliosis [14, 15], was used for structural data collection at each visit. The CCS was performed early during hospitalization, typically within the first 24–48 h. The CCS comprises 39 items which measure 17 subjective symptoms and 22 objective neurological findings from peripheral nervous system (PNS) and CNS. Each item is scored as 0 = none, 1 = mild (no influence on daily life) or 2 = serious (with influence on daily life), consequently the sum score ranged from 0 to 78. The sum of scores for symptoms, objective findings, and the total score were recorded. Additional information is available in a previous publication [11]. At the follow-up visits, only symptoms and objective findings that were newly developed or worsened since the onset of TBE, and which had no other obvious medical explanation, were scored. Unclear issues were discussed within the study group to achieve consensus.

### Patient-reported outcome measures (PROMs)

We selected RAND 36-item short form health survey (RAND-36) [16] to assess health-related quality of life due to its cost-free availability. The results from RAND-36 at 12 months were compared with normative data from the Norwegian population based on the Short Form (SF)−36 Health Survey [17]. The SF-36 assessment of the Norwegian reference population was conducted using a sample randomly selected from the National Population Register, achieving a response rate of 67% (2323 individuals). From this reference group, we used crude scores and scores adjusted for sex-, age- and educational level [18]. The questions in RAND-36 and SF-36 are identical comprising 36 items grouped into eight scales: physical functioning, physical role limitations, emotional role limitations, vitality, mental health, bodily pain, social functioning and general health. The scales are aggregated into two summary scores, the Physical and Mental Component Summary scores: Each item is scored from 1 to 100, with higher scores indicating a more favorable health status. The RAND-36’s domains were scored and calculated according to the SF-36 Health Survey Manual and Interpretation Guide [19]. We used Fatigue Severity Scale (FSS) to assess fatigue at all study points [20]. FSS consists of nine items addressing the physical, social and cognitive effects of fatigue. Each item is scored on a Likert scale ranging from 1 to 7, with 1 representing complete disagreement and 7 complete agreements. The item scores are summed and divided by nine, and higher score indicates greater levels of fatigue, while a mean score ≥ 4 are considered severe fatigue [21].

### Statistical methods

Continuous variables were tested for normality and means, standard deviations (SD), medians, interquartile ranges (IQR) and ranges were reported as appropriate. Categorical data were reported as frequencies and percentages. We used Spearman`s rank correlation to examine correlation between two non-normally distributed variables. Comparison tests such as *t*-tests, Wilcoxon rank sum test, or Kruskal Wallis test, were performed by Dunn test with Bonferroni correction as appropriate. Dependent samples t-test or Wilcoxon signed rank test were used to assess the difference between paired measurements from baseline to follow-ups. We used bootstrap estimation with 10,000 repetitions to calculate percent change and confidence intervals in CCS from baseline to 3-month follow-up and from baseline to 12-month follow-up. Pearson’s chi-squared or Fisher’s exact tests were used to test the differences in proportions for independent categorical variables, while McNemar`s test was used for dependent variables involving a dichotomous variable. Statistical significance was defined as a level of *p* < 0.05. Based on results from a previous study [22], we considered a difference of 7% (one-half standard deviation) in the SF-36 physical and mental summary scores between the TBE group and the national reference group [17] to be clinically significant. We performed a sensitivity analysis by excluding patients with missing data, and since the results remained unchanged, imputation was not performed. We used the statistical program STATA (StataCorp. 2023. *Stata Statistical Software: Release 18*. College Station, TX: StataCorp LLC) in analysing the data.

### Ethical considerations

All patients gave written informed consent before inclusion. The study was approved by the Regional Ethics Committee in South-East Norway (Study no 96505) and by the data protection officers at each participating hospital. The trial was registered at clinicaltrials.gov (ID: NCT05138055).

## Results

### Patient characteristics

**Fig. 1:**
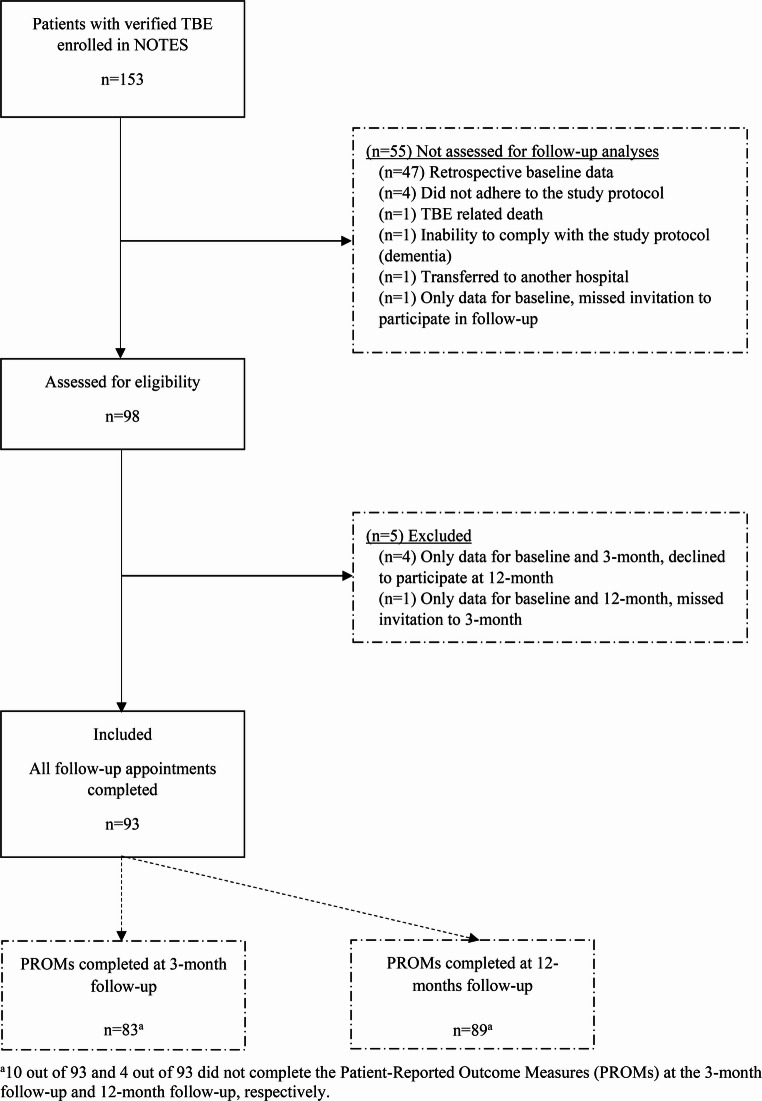
Study flow chart

Of the 153 patients included in NOTES` publication of baseline characteristics [11], 98 were eligible for this follow-up study, and 93 were included in the analyses. Details are shown in the flowchart (Fig. 1). Baseline characteristics are presented in Table 1.

**Table 1:** Baseline characteristics

Characteristics	TBE *n* = 93
Demographics	
Age at admission mean (SD)	54.6 (16.2)
Age groups	
16–30	7 (7.5)
31–45	19 (20.4)
46–60	35 (37.6)
>60	32 (34.4)
Gender	
Female	37 (39.8)
Male	56 (60.2)
Living alone	
Yes	12 (12.9)
Level of education ^a^	
Education ≤ 12/13 years	44 (47.3)
Education > 12/13 years (University/college)	47 (50.4)
Missing	2 (2.2)
Occupational status at admission	
Full-time job/part-time job/student	56 (60.2)
Age pensioner	22 (23.7)
Unemployed/sick leave/disability	14(15.1)
Missing	1 (1.1)
Health Status and Comorbidities	
Any comorbidity, yes	47 (50.4)
Immunosuppression^b^	8 (8.6)
TBE-related characteristics	
Known tick bite	49 (52.7)
Symptom onset^c^ to admission, days, median (IQR)	14 (8.5–14)
Length of stay, median (IQR)	7 (4–10)
Monophasic course	40 (43.0)
Vaccinated against TBE^d^	5 (5.4)
Disease severity	
Mild	23 (24.7)
Moderate	59 (63.4)
Severe	11 (11.8)
Medical Care	
Treated in intensive care unit	5 (5.4)
Required mechanical ventilation	3 (3.2)
Treated in rehabilitation institution^e^	13 (14.0)

Data are given as *n* (%) unless otherwise stated

^a^ Categorization of education level was based on the Norwegian educational system and was dichotomized as (a) education for up to 12 years (or 13 years from 1994 for those who were born in 1981 and above) (b) education exceeding 12 (or 13) years as higher education referring to university or college degrees

^b^ Immunosuppression was defined as active cancer, both hematologic malignancy and solid tumors, other severe chronic disease or immunosuppressive conditions, including primary immunodeficiencies, HIV infection, organ transplantation and cytostatic or other immunosuppressive treatment

^c^ The number of days from symptom onset to admission was counted from the first day of symptoms in the first phase

^d^ All incomplete vaccinated according to national guidelines

^e^ Only inpatients registered

Among the 75 patients of working age (≤ 67 years), 56/75 (74.7%) were actively employed, including three students. Prior to TBE, all patients were managing their lives independently. Noteworthy, over 40% of the patients experienced a monophasic course. About half of the patients had comorbidities, the most common being hypertension (*n* = 14, 15.0%), allergies (*n* = 11, 12.8%), and chronic heart disease (*n* = 10, 10.8%). During the hospital stay for TBE, 70 (75.3%) patients were categorized with moderate or severe disease (Table 1). EEG was carried out in 50 (53.7%) patients and revelated abnormalities suggestive of encephalitis in 38 (76.0%). At baseline, all patients reported symptoms and/or presented objective findings, including 91 (97.8%) patients who experienced impacts on their daily life (CCS score of 2). Of the 13 patients needing rehabilitation after hospitalization, eight had severe disease; the median age was 65, with four patients under 50 at admission.

### Follow-up of tick-borne encephalitis

**Fig. 2:**
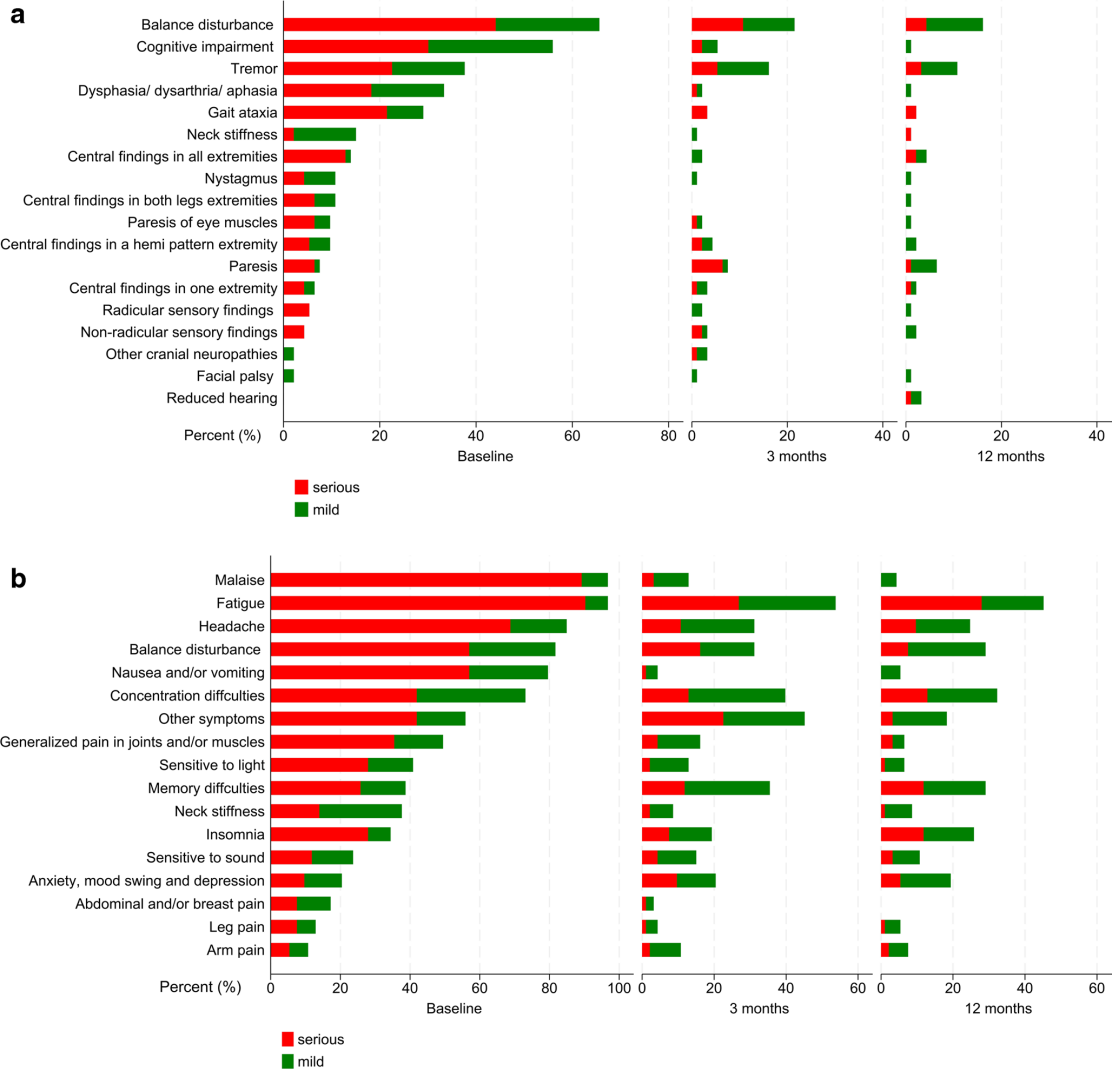
**A** Objective findings as scored on the composite clinical score variables at baseline, 3-month, and 12-month follow-ups. **B** Symptoms as scored on the composite clinical score variables at baseline, 3-month, and 12-month follow-ups

Of the 56 active workers, data were available for 50 at 3-month follow-up. Among these, 41 (82.0%) required part-time or full-time sick leave during the first three months, while nine did not; the reasons were not recorded. Six had missing data. Between 3 and 12 months, 25 out of 56 (44.6%) had sick leave, though exact duration and extent were not documented. By the 12-month follow-up, 4 out of 56 (7.1%) remained on full-time sick leave. The first follow-up visit occurred at a median of three months after admission, though one patient was seen at six months and two at five months. The last follow-up was at a median of 12 months (range 10 to 14 months) after admission. The course of findings and symptoms during follow-up is shown in Fig. 2A and B and Supplementary Table 1.

### 3-month follow-up: objective findings and symptoms

Overall, three months after admission, 77 (82.8%) patients reported symptoms and/or presented objective findings, including 48 (51.5%) patients who experienced impacts on their daily life with CCS score of 2 (Fig. 2).

Among 42 (45.1%) patients with objective neurological findings, 21 (22.6%) had findings that influenced their daily life.

The most common were impaired balance in 20 patients (21.5%) and tremor in 15 (16.1%). Eight (8.6%) patients had findings in the extremities indicative of CNS involvement, characterized by positive plantar reflex, hyperreflexia, spasticity/hypertonia, slower speed and/or impaired coordination. Seven (7.5%) patients were diagnosed with paresis of the extremities. Moreover, five (5.4%) patients presented with cognitive impairment, three assessed through objective neuropsychological tests which were not part of the study protocol, and two observed by slow thinking during consultations. Of these patients, three were classified with severe disease at the time of admission, requiring intensive care unit treatment, and prolonged inpatients rehabilitation during the first three months. This impairment was obvious at the first follow-up, but it was reversible for all but one woman in her 70s.

The most reported symptoms were fatigue in 50 (53.7%) cases, concentration difficulties in 37 (39.8%) cases, memory issues in 33 (35.4%) cases, both headaches and balance problems in 29 (33.3%) cases each, and sleep disturbances in 24 (25.8%) cases. Out of 42 (45.1%) patients who reported symptoms not directly addressed in the questionnaire, hair loss was the most common, affecting 23 (24.7%).

### 12-month follow-up: objective findings and symptoms

Overall, one year after TBE, 69 (74.2%) patients reported symptoms and/or presented objective findings. Of these, 38 (40.9%) patients experienced impacts on their daily life indicated by a CCS score of 2 (Fig. 2).

Among the 31 (33.3%) patients who had objective neurological findings, seven (7.5%) patients reported an impact on their daily life.

The most common objective findings were balance disturbances in 15 (16.1%) patients, tremor in 10 (10.8%), and central extremity findings and paresis in six each (6.5%). Among the seven patients who had paresis in the extremities at 3 months, one achieved complete recovery. The other five patients experienced a decline in severity from severe to mild paresis. One severely ill patient in his forties without comorbidities maintained severe paresis throughout the entire year. Hearing loss, which had not been noted during the earlier study points, were identified in three patients, of whom two were under 45 years. Of the two patients with central facial palsy during the acute phase, one patient had persisting findings at 12 months.

In total, 66 (71.0%) patients reported symptoms one year after TBE, with 36 patients (38.7%) reporting an impact on daily life.

The most prevalent symptoms affecting daily life were fatigue in 26 cases (28%), followed by concentration difficulties in 12 cases (13%), and memory issues and sleep disturbances, each affecting 11 (11.8%) cases. There was no significant relationship between memory problems and disease severity. Among those with memory disturbances, all but two were active workers, with a median age of 55 years. Individuals reporting sleep problems were more likely to also report fatigue (*p* < 0.001). Nine cases (10%) reported headaches, with all but one being under 60 years old; six were women. Additionally, seven cases (8%) experienced balance problems, as confirmed by balance tests, and five of these individuals were over 60 years old. The hair loss reported at the 3-month follow-up had improved. One male in his early 60 s reported reduced libido, a symptom not assessed in the questionnaire.

### Changes in composite clinical score from baseline to 3- and 12-month follow-ups

**Fig. 3:**
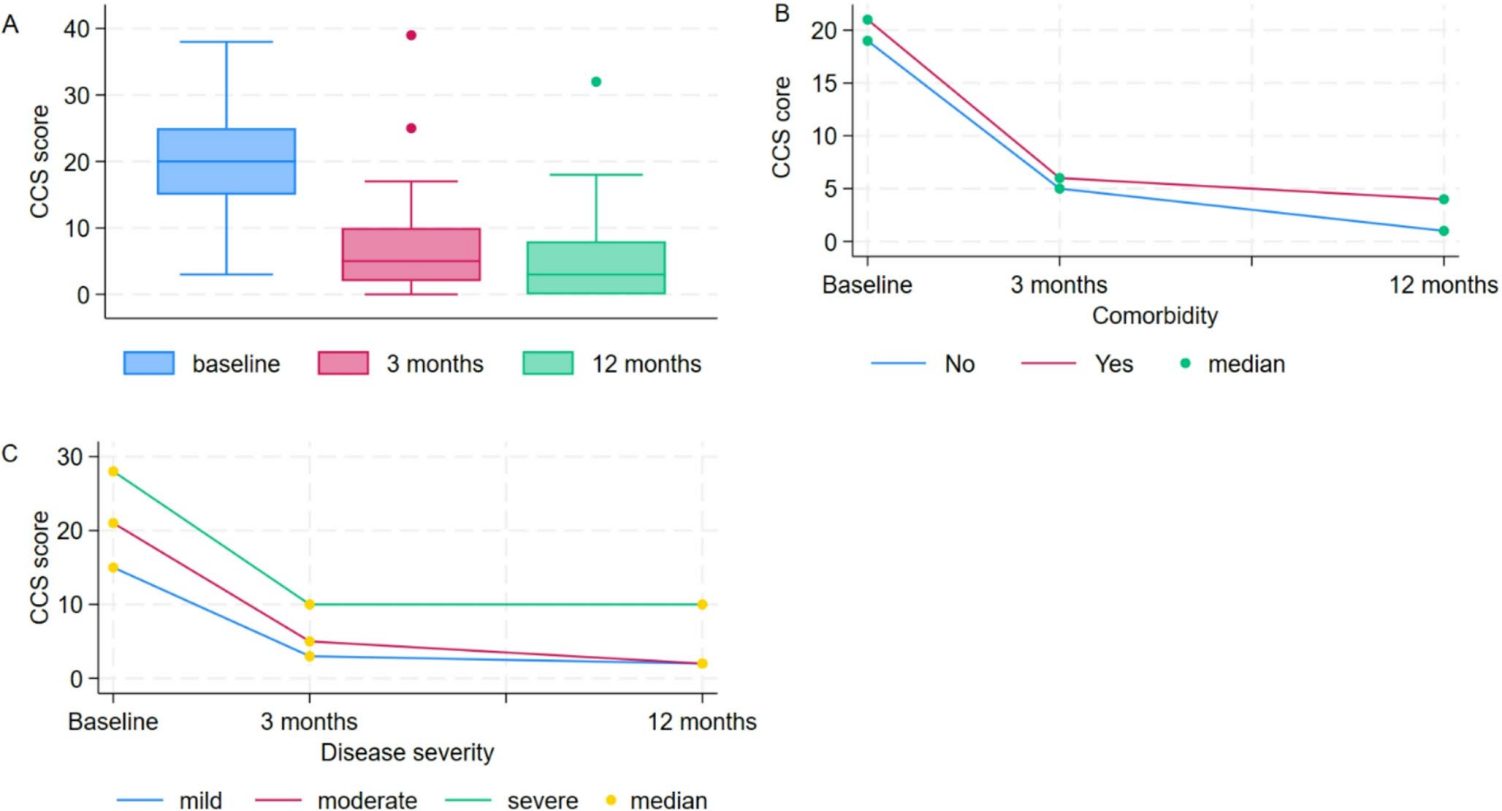
Changes in composite clinical Score (CCS) from baseline to 3- and 12-month follow-ups, stratified by (**A**) CCS sum score and (**B**) comorbidity and (**C**) disease severity

The CCS sum score decreased significantly from baseline to both visits for the whole group, although most of the improvement was observed from baseline to 3 months (*p* < 0.001, Fig. 3A). Patients with comorbidities differed from those without comorbidities at both visits (*p* = 0.048, *p* = 0.015, Fig. 3B). Those classified with mild disease showed a decrease in CCS sum score from baseline to 3 months (*p* < 0.001), but not from 3- to 12-month. At both visits, there was a difference in CCS sum score between patients with severe diseases compared to those with moderate and mild disease (Fig. 3C). At 12-month follow-up there were no differences in CCS sum score between those with mild and moderate disease (Fig. 3C).

For the entire study population, the improvement from baseline to 3-month follow-up was 68.4% and reached 77.2% from baseline to 12-month follow-up. Patients with moderate disease improved 80.4%, and those with severe diseases 68.4% from baseline to 12-month follow-up. Patients with and without comorbidities improved slightly above 70% and 80% in the first year. Patients under 30 years of age tended to have greater improvement compared to older age groups, with an improvement of over 90% during the first year. The CCS scores for different variables with p-values and percent change are further described in Supplementary Tables 2 and Table 4.

### Health-related quality of life at 12-months follow-up

**Fig. 4:**
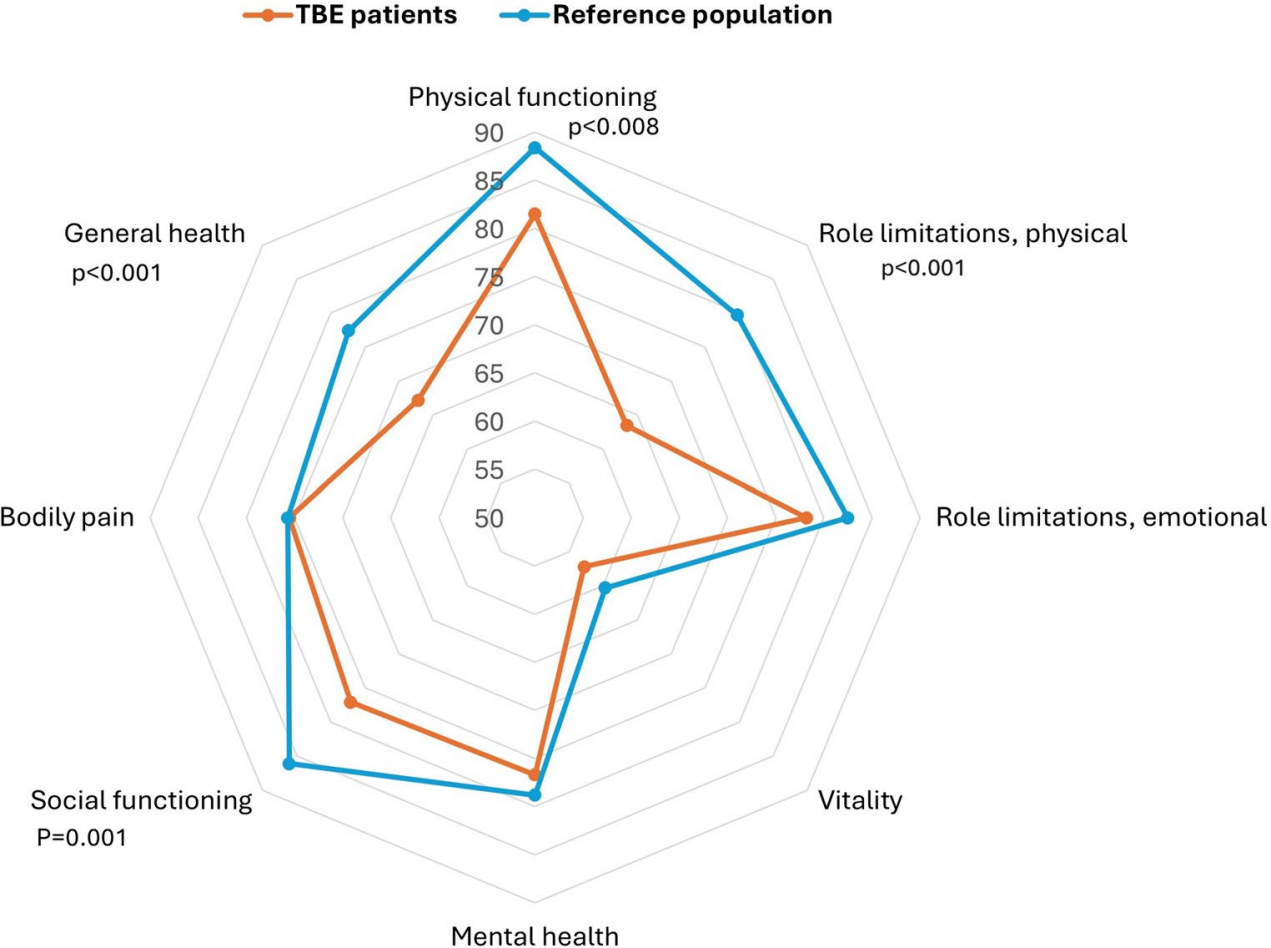
Comparison of RAND-36 domain scores between TBE patients at 12 months and SF-36 results for the general Norwegian population. Significant differences are noted. Higher score indicates greater quality of life

To assess health-related quality of life one year after acute illness, the RAND-36 questionnaire, completed by 89 TBE patients, was compared with normative data from 2214 controls [18]. The controls had higher mean scores than the patients in four of eight individual domains: physical functioning, role limitations due to physical health, social functioning, and general health perception (Fig. 4 and Supplementary Table 4). Cohen’s d for these domains ranged from 0.39 to 0.47. Adjusting for age, sex and the level of education, did not alter the results. Patients with severe disease had the lowest scores in all scales (data not shown), but the differences did not reach statistical significance compared to the reference group. For patients with mild disease, the only observed difference compared to the reference group was in the general health domain (*p* = 0.0144). Women in the cohort showed results like the entire TBE-group, whereas men varied from the reference group in social functioning and general health. Patients with comorbidities exhibited differences in the same four domains, whereas those without comorbidities showed significant differences in the domain of role limitations due to physical health (*p* = 0.044). No significant differences were found between patients with monophasic and biphasic course.

### Fatigue severity scale at 12-Month Follow-Up

Fatigue, the most reported symptom one year after TBE, significantly correlated with answers to the FSS-questionnaire (*p* < 0.001). The median FSS score for those reporting fatigue was 5.0 (IQR 4.1–6.3). Patients with severe fatigue (FSS score ≥ 4) scored significantly lower (*p* < 0.001) in all RAND-36 domains compared to those without fatigue (FSS score < 4), (Supplementary Table 5). Age, disease severity in the acute phase, gender, living alone, comorbidity and level of education did not influence the results. Patients with comorbidities had a higher level of FSS after one year, though not statistically significant (*p* = 0.08). FSS scores decreased from baseline to 3-months (*p* < 0.001), but not from 3 to 12 months follow-ups for the entire cohort (Supplementary Table 1).

## Discussion

In this TBE cohort, most improvements occurred within the first three months, with an overall CCS sum score reduction of nearly 70%. Although there was an 80% improvement in the first year, 41% of patients still report ongoing complaints affecting daily life after one year. Self-reported symptoms constituted 39% of these complaints, with fatigue, memory difficulties, concentration issues, and sleep disturbances being most common. Clinical investigations showed that 8% had objective findings impacting daily life, predominantly balance disturbances and tremors. Additionally, 14% required inpatient rehabilitation after discharge, and over 7% of active workers were on full-time sick leave related to TBE after one year. These findings illustrate that TBE affects not only the individual recovery but also imposes a social burden, resulting in economic consequences from reduced workability and the costs associated with rehabilitation. Comorbidities and severe disease negatively impact long-term outcomes as indicated by higher CCS sum scores at 12-month follow-up. TBE patients also reported lower health-related quality of life, particularly regarding physical health, compared to the general population in Norway.

The distribution of sex and age aligns with previous studies [9, 23–25] showing a dominance of middle-aged individuals, with slight predominance of men. Mild TBE in the acute phase was observed in nearly 25% of patients, consistent with other European studies [7, 8] while research from Sweden [26] and Lithuania [9] reported mild disease in 45% and 53% of cases, respectively. We find no clear explanation for this difference, as definitions of severity and patient inclusion rates are comparable. Positive EEG results exclude mild cases based on our study’s disease classification. The current study’s EEG performance is higher (54%), than two other studies (33% and 25%) with comparable disease severity definitions [27, 28], which may influence the results. Larger studies incorporating EEG are needed to confirm these findings, as mild cases may be misclassified.

Monophasic course was observed in 43% of the patients, aligning with two previous studies [24, 29]. However, other prospective studies reported notably lower proportions of monophasic course [9, 25, 27, 30]. Monophasic course has been suggested to indicate more severe disease [29], and notably, the Far Eastern subtype, associated with the highest mortality rate, causes a monophasic course [31]. In the current study, the monophasic course was not associated with unfavorable outcomes one year after TBE, supporting findings from other European studies [29, 32]. Whether the difference in course and severity may be due to varying virus strains, selection bias, or differences in the patients` immune responses needs further investigation.

The proportion of patients with lasting complains in this study aligns with findings from other European cohorts. Specifically, 41% of patients had persistent problems affecting daily life one year after infection, which is comparable to a study showing that 36% of adult patients experienced complaints after 1.5 years [8]. Likewise, the 8% of patients with objective findings impacting daily life after one year is consistent with a Slovenian study reporting 10% with at least one objective sign at 12-month follow-up [7]. In contrast, a Lithuanian study from 2024 found that 39% of patients still had objective neurological signs 1.5 years after TBE [9]. When including patients with mild findings (e.g., facial palsy detectable only on investigation), the percentage of objective findings in our study increases to 33% at one year, making it comparable to the Lithuanian study.

In our study, 14% of patients received rehabilitation in an institutional setting. Interestingly, this proportion is lower than what has been reported in other European studies. A Lithuanian study found that 30% of patients received institutional rehabilitation [9], while a German study reported that 33% of patients received rehabilitation, with more than half of those aged over 65 [23]. Notably, the German study included both inpatient and outpatient rehabilitation services. In Norway, rehabilitation after tick-borne diseases is poorly specialized and not systematically implemented [33]. The OECD report *Sickness and Disability Systems* highlights limited involvement from employers and general practitioners, weak coordination between services, and a lack of incentives to support return to work in the Norwegian system [34]. These factors may explain the lower rehabilitation rate among Norwegian TBE patients.

Overall, the frequency and severity of objective findings and symptoms decreased from baseline to 3-month follow-ups and from 3 to 12-months (*p* < 0.001). After one year, main objective findings included impaired balance and tremors, along with symptoms of fatigue, concentration difficulties, and memory and sleep problems, consistent with previous studies [7–9]. The finding that six out of seven patients still had extremity paresis supports previous studies indicating the long-term persistence of paresis [7, 9]. This indicates that, while physical impairments were captured, cognitive and psychological challenges also persist after TBE, highlighting its multifaceted impact.

Of those reporting memory difficulties, 67% were of working age. We assume that active workers may recognize memory issues more readily due to greater life demands, leading to more frequent reports than non-working patients. Interestingly, a recent study [9] evaluated memory loss after TBE with neuropsychological tests, found that subjective memory difficulties were related to slower processing speed rather than actual memory decline. Nonetheless, patients report memory disturbances as a long-term sequela in several studies [7, 8, 28, 30, 35].

Notably, hair loss—a reversible symptom—affected nearly one in four patients during the first three months, consistent with findings from a 1999 study on a TBE cohort [36].

Having comorbidities and severe disease were associated with unfavorable outcomes, supporting the findings from a German study [8]. Patients with mild disease had better outcomes than patients with moderate diseases at 3-month follow-up, but by 12 months, both groups showed comparable recovery levels. Interestingly, Veje et al. found that long-term memory impairment occurred regardless of TBE severity [28]. This suggests that acute disease severity may not fully predict long-term outcomes, particularly for cognitive function. Supporting this, Griska et al. reported neurological sequelae persisted for 1.5 years after TBE, even in cases that were initially classified as mild [9]. In contrast, Quist-Paulsen et al. [37] found no neuropsychological deficits 12 months after discharge in patients with aseptic meningitis, which is comparable with mild TBE. However, none of their patients were infected by TBEV.

TBE patients showed a statistically significant reduction in four of the eight RAND-36 dimensions compared to the reference population, with moderate effects indicated by Cohen’s d values. Interestingly, reductions occurred in three physical health domains, while the only mental health domain where TBE patients scored lower was social functioning. This aligns with a recent study that found significant improvement in six out of seven neurocognitive domains from six to 18 months, except for social cognition [9], suggesting that difficulty in interpreting social cues may hinder effective interactions with others, leading to lower social functioning. Additionally, Veje et al. demonstrated unfavorable social outcomes in TBE patients [28]. Patients with severe fatigue scored significantly lower in all RAND-36 domains compared to those with FSS score < 4, indicating that severe fatigue negatively impacts health-related quality of life. Furthermore, fatigue was associated with sleep disturbances in the current study, supporting a review linking TBE to sleep-wake sequelae that may contribute to increased fatigue [38]. Veje et al. also reported greater self-assessed daytime sleepiness and fatigue in TBE patients compared to control group [39].

A major strength of our study is the well-defined, population-based cohort with a one-year longitudinal follow-up and minimal dropouts, achieving a 95% inclusion rate for eligible patients. Only five eligible patients were excluded, minimizing potential bias from missing follow-up information. Conducted in endemic TBE regions, our study included all three hospitals responsible for most TBE admissions in Norway, making our findings representative of adult TBE cases in the country.

The current study has a limitation due to the lack of objective neuropsychological tests. Future research should include such tests to better assess cognitive function. Cognitive decline can be subtle, appearing only in complex daily life, making detection difficult. Therefore, selecting appropriate tests is crucial, as studies suggest younger, highly educated patients may compensate for cognitive decline during assessments [40].

Another limitation of this study is the small sample size, which increases the risk of Type II errors. Therefore, we could not find statistically significant differences in health-related quality of life between patients with severe disease and the reference population, despite markedly lower scores compared to those with moderate and mild disease. Although patients under 30 showed the greatest improvement, this was not significantly different from older age groups, likely due to Type II errors from the limited sample size.

We used a modified composite clinical score, originally designed for neuroborreliosis, to record clinical features. TBEV affects the CNS; thus, distinguishing between PNS- and CNS-findings is irrelevant for many characteristics. Notably, five of six patients with paresis, a peripheral finding according to CCS, also showed other CNS findings after one year, suggesting that the classification into peripheral and central paresis may be inaccurate. Consequently, we did not differentiate between peripheral and central findings in the results. Recently, an expert panel highlighted the need for development of a classification system that captures the acute phase and the outcomes of TBE [41]. We strongly support this, as it would facilitate information collection and analysis across Europe.

We chose a one-year follow-up period, which provided a practical timeframe for data collection and was more resource-efficient than longer studies. This choice is supported by a Slovenian study showing that post-encephalitic syndrome stabilized 12 months after the acute illness [7]. However, a limitation is that we cannot assess whether symptoms or objective findings continue to diminish after 12 months or if some individuals have learned to cope with the sequelae.

## Conclusion

Our findings show that clinical improvement for the entire TBE-cohort exceeds 80% during the first year. Nevertheless, a notable portion—over 40%—of patients continue to experience complaints one year after TBE, which may affect their ability to work. The most common self-reported symptoms were fatigue and difficulties with concentration, memory, and sleep, while clinical investigations revealed a predominance of impaired balance and tremor. Comorbidities and severe disease negatively affect long-term outcomes. Furthermore, TBE patients report a lower health-related quality of life one year after admission, particularly regarding physical health, compared to the general population. Our study underscores that TBE is a serious neurological illness affecting individuals in endemic areas, highlighting the need for preventive vaccination. Importantly, the persistence of symptoms and signs in a substantial proportion of patients reveals unmet needs for structured rehabilitation programs, addressing both physical and neuropsychological aspects in recovery. Moreover, a standardized case record form is needed across Europe to facilitate comparison of TBE cases.

## Supplementary Information

Below is the link to the electronic supplementary material.

Supplementary Material 1

## Data Availability

The datasets generated during and/or analyzed during the current study are not publicly available because the study is still ongoing; however, they are available from the corresponding author upon reasonable request. The data were handled in compliance with the General Data Protection Regulation (GDRP) requirements. The data are stored on TSD (Tjeneste for Sensitive Data) facilities owned by the University of Oslo, operated, and developed by the TSD service group at the University of Oslo, IT-Department (USIT) ([tsd-drift@usit.uio](mailto: tsd-drift@usit.uio)).
