# Supplementary material for: Tick-borne encephalitis in Norway: A cohort study of clinical course and health-related quality of life at three- and twelve-month follow-up
Source: Eur J Clin Microbiol Infect Dis. 2025 Nov 8;45(2):567–79. doi: 10.1007/s10096-025-05341-z (PMC12987897; doi:10.1007/s10096-025-05341-z)
Supplement: Supplementary file 1 — Supplementary Material 1 [file 10096_2025_5341_MOESM1_ESM.docx]

**Supplementary information on Composite clinical score and PROMs**

**Table 1** Changes in clinical characteristics and PROMs from baseline to 3- and 12-month follow-ups

|  | | **Baseline** | **3 months** | **12 months** | **p^a^** | **p^b^** |
| --- | --- | --- | --- | --- | --- | --- |
| **Composite Clinical Score, n= 93** | |  | | | | |
| Total score, median (IQR) | | 20 (15-25) | 5 (2-10) | 3 (0-8) | <0.001 | <0.001 |
| Symptom score, median (IQR) | | 15 (12-18) | 4 (2-7) | 2 (0-7) | <0.001 | <0.001 |
| Objective findings, median (IQR) | | 4 (1-8) | 0 (0-2) | 0 (0-1) | <0.001 | <0.001 |
| ≥1 any variable scored 2 | | 91 | 48 | 38 | - | - |
| ≥1 objective variable scored 2 | | 58 | 21 | 7 | - | - |
| ≥1 symptom variable scored 2 | | 91 | 47 | 36 | **-** | **-** |
| **Clinical characteristics, n= 93** | |  |  |  |  |  |
| Balance, positive test | | 61 (65.6) | 20 (20.5) | 15 (16.1) | **-** | **-** |
| Tremor | | 35 (37.3) | 15 (16.1) | 10 (10.8) | **-** | **-** |
| Central findings affecting  extremities^c^ | | 42 (45.2) | 8 (8.6) | 6 (6.5) | **-** | **-** |
| Ataxia | | 27 (29.3) | 3 (3.2) | 2 (2.1) | **-** | **-** |
| Paresis, extremities^d^ | | 7 (7.5) | 7 (7.5) | 6 (6.5) | **-** | **-** |
| Facial palcy (all central) | | 2 (2.2) | 1 (1.2) | 1 (1.2) | **-** | **-** |
| **PROMs** | |  |  |  |  |  |
| **RAND-36**  Summary scores | | n= 68 | n= 81 | n= 82 |  |  |
| PCS | mean (SD) | 29.9 (9.0) | 44.4 (10.9) | 46.9 (11.1) | <0.001 | 0.004 |
| MCS | mean (SD) | 42.3 (11.5) | 47.9 (11.5) | 51.0 (10.5) | 0.001 | 0.004 |
| **Fatigue severity scale, (FSS)** | | n= 68 | n= 82 | n= 82 |  |  |
| FSS, median (range) | | 6.4 (1.9-7) | 3.7 (1-7) | 3.2 (1-7) | <0.001 | 0.218 |
| FSS $\geq$4 n (%) | | 58 (85.3) | 38 (46.3) | 33 (40.2) | <0.001 | 0.225 |

Higher CCS indicates more symptoms and/or objective findings; Statistical significance at the level p<0.05 is shown in red text

^a^ Change in scores from baseline to 3 months

^b^ Change in scores from 3 months to 12 months

^c^ Including a positive plantar reflex, hyperreflexia, spasticity/hypertonia, slower speed or impaired coordination

^d^ Lumbar, cervical or non-radicular paresis

**Table 2** Composite Clinical Score of TBE patients over time across various characteristics (n=93) with median and IQR

| **Characteristics** | **Composite Clinical Score (CCS)**  Median (IQR) | | | **p (baseline vs 3)** | **p (3 vs 12)** |
| --- | --- | --- | --- | --- | --- |
|  | **baseline** | **3 months** | **12 months** |  |  |
| **CCS, n= 93** | 20 (15-25) | 5 (2-10) | 3 (0-8) | <0.001 | <0.001 |
| **Age group** |  |  |  |  |  |
| 16-30 | 22 (20-24) | 4 (0-9) | 1 (0-3) | 0.016 | 0.125 |
| 31-45 | 20 (17-26) | 6 (1-13) | 4 (0-10) | <0.001 | 0.063 |
| 46-60 | 19 (13-25) | 4 (2-11) | 3 (0-9) | <0.001 | 0.074 |
| >60 | 20 (15-24) | 5 (2-9) | 3 (1-7) | <0.001 | 0.001 |
| **p** | 0.729 | 0.753 | 0.410 |  |  |
| **Gender** |  |  |  |  |  |
| Male | 21 (15-25) | 4 (1-10) | 2 (0-6) | <0.001 | 0.003 |
| Female | 20 (15-24) | 6 (3-11) | 3 (1-9) | <0.001 | 0.013 |
| **p** | 0.970 | 0.098 | 0.241 |  |  |
| **Education** |  |  |  |  |  |
| Education ≤ 12/13 years | 20 (15-25) | 5 (1-10) | 3 (1-7) | <0.001 | 0.005 |
| Education > 12/13 years | 20 (15-25) | 5 (2-10) | 3 (0-9) | <0.001 | 0.003 |
| **p** | 0.789 | 0.624 | 0.638 |  |  |
| **Living alone** |  |  |  |  |  |
| Yes | 19 (14-26) | 5 (2-11) | 6 (1-12) | <0.001 | 1.000 |
| No | 20 (15-24) | 5 (2-10) | 3 (0-7) | <0.001 | <0.001 |
| **p** | 0.652 | 0.994 | 0.310 |  |  |
| **Any comorbidity** |  |  |  |  |  |
| Yes | 21 (15-24) | 6 (3-10) | 4 (1-9) | <0.001 | 0.003 |
| No | 19 (15-25) | 5 (0-9) | 1 (0-6) | <0.001 | 0.008 |
| **p** | 0.944 | 0.048 | 0.015 |  |  |
| **Hyponatremia** |  |  |  |  |  |
| Yes | 21 (15-28) | 5 (2-10) | 3 (0-9) | <0.001 | <0.001 |
| No | 19 (13-23) | 6 (1-10) | 3 (0-8) | <0.001 | 0.043 |
| **p** | 0.053 | 0.578 | 0.620 |  |  |
| **Biphasic course** |  |  |  |  |  |
| Yes | 20 (15-25) | 5 (2-10) | 2 (0-8) | <0.001 | <0.001 |
| No | 20 (15-24) | 4 (2-10) | 3 (1-9) | <0.001 | 0.029 |
| **p** | 0.776 | 0.812 | 0.676 |  |  |
| **Pleocytosis** |  |  |  |  |  |
| Yes | 21 (15-25) | 5 (1-10) | 3 (0-8) | <0.001 | <0.001 |
| No | 18 (6-20) | 10 (2-12) | 3 (2-10) | 0.141 | 0.250 |
| **p** | 0.203 | 0.286 | 0.464 |  |  |
| **Severity of diseases** |  |  |  |  |  |
| Mild | 15 (8-19) | 3 (0-6) | 2 (0-7) | <0.001 | 0.329 |
| Moderate | 21 (16-25) | 5 (1-9) | 2 (0-6) | <0.001 | <0.001 |
| Severe | 28 (22-34) | 10 (10-12) | 10 (5-11) | 0.001 | 0.043 |
| **p^a^** | <0.001 | 0.001 | 0.007 |  |  |

IQR=interquartile range; Statistical significance at the level p<0.05 is shown in red text

^a^Comparison test with Bonferroni correction: Severity of diseases: **at baseline**, mild vs moderate, adjusted p <0.001; mild vs severe, adjusted p < 0.001; moderate vs severe, adjusted p = 0.031; **at 3 months**, mild vs moderate, adjusted p = 0.269; mild vs severe, adjusted p < 0.001; moderate vs severe, adjusted p = 0.002; **at 12 months**, mild vs moderate, adjusted p > 0.999; mild vs severe, adjusted p = 0.008; moderate vs severe, adjusted p = 0.003.

**Table 3** Composite Clinical Score (CCS) of TBE patients over time across various characteristics (n=93) with mean and SD

Percent Change from Baseline to 3 months and from Baseline to 12 months with 95% Confidence Interval

| **Characteristics** | **Composite Clinical Score (CCS),**  Mean, (SD) | | | **% change CCS^a^** | **(95% CI)** | **% change CCS^b^** | **(95% CI)** |
| --- | --- | --- | --- | --- | --- | --- | --- |
|  | **baseline** | **3 months** | **12 months** |  |  |  |  |
| **CCS,** n= 93 | 20.0 (7.6) | 6.3 (6.1) | 4.6 (5.4) | 68.4% | (63-74) | 77.2% | (72– 82) |
| **By age group, years** | | | | | | | |
| 16-30, n= 7 | 21.6 (3.0) | 4.4 (4.3) | 2.0 (3.3) | 79.5% | (68- 92) | 90.7% | (80– 99) |
| 31-45, n= 19 | 20.9 (6.5) | 8.0 (9.0) | 5.9 (7.7) | 61.8% | (43– 77) | 71.6% | (55– 85) |
| 46-60, n= 35 | 19.5 (8.6) | 6.0 (5.0) | 4.9 (4.8) | 69.2% | (61– 77) | 75.1% | (68 – 82) |
| >60, n= 32 | 19.7 (7.9) | 6.1 (5.5) | 4.0 (4.5) | 69.1% | (61– 77) | 79.9% | (73– 86) |
| **By gender** |  |  |  |  |  |  |  |
| Male, n=56 | 20.0 (8.1) | 5.7 (6.3) | 4.1 (5.5) | 71.4% | (63– 78) | 79.2% | (72– 85) |
| Female, n=37 | 20.1 (6.9) | 7.2 (5.7) | 5.1 (5.1) | 68.4% | (63– 74) | 74.2% | (67– 81) |
| **By education, n=91** | |  |  |  |  |  |  |
| Education ≤ 12/13 y, n=44 | 20.2 (8.1) | 6.0 (5.6) | 4.2 (4.7) | 70.1% | (63- 77) | 79.2% | (73– 85) |
| Education > 12/13 y, n=47 | 20.3 (6.9) | 6.7 (6.6) | 5.0 (6.0) | 66.8% | (58- 75) | 75.2% | (69- 82) |
| **By living alone** |  |  |  |  |  |  |  |
| Yes, n= 12 | 18.8 (6.9) | 5.8 (4.7) | 6.2 (5.8) | 68.9% | (55- 82) | 67.1% | (52- 82) |
| No, n= 81 | 20.2 (7.7) | 56.4 (6.3) | 4.3 (5.3) | 68.4% | (62- 74) | 78.6% | (73- 83) |
| **By comorbidity** |  |  |  |  |  |  |  |
| Yes, n=47 | 19.9 (8.1) | 7.0 (5.0) | 5.2 (4.7) | 65.0% | (59- 71) | 73.4% | (68- 79) |
| No, n=46 | 20.1 (7.1) | 5.6 (7.1) | 3.8 (5.9) | 71.9% | (62- 80) | 81.1% | (73- 88) |
| **By biphasic course, n=92** | | | | | | | |
| Yes, n=52 | 20.0 (7.4) | 6.4 (6.5) | 4.5 (5.6) | 67.9% | (59- 75) | 77.4% | (69- 84) |
| No, n=40 | 19.9 (7.9) | 6.0 (5.5) | 4.7 (5.0) | 70.1% | (62- 77) | 76.2% | (70- 83) |
| **By pleocytosis, n=89** | |  |  |  |  |  |  |
| Yes, n= 82 | 20.4 (7.4) | 6.2 (6.3) | 4.5 (5.4) | 70.0% | (64- 75) | 78.0% | (73- 83) |
| No, n= 7 | 17.5 (7.5) | 6.2 (3.3) | 4.8 (5.7) | 51.1% | (17- 74) | 70.4% | (43- 88) |
| **By severity** |  |  |  |  |  |  |  |
| Mild, n=23 | 14.1 (6.9) | 4.1 (4.0) | 3.9 (4.4) | 70.8% | (58- 81) | 73.2% | (61- 84) |
| Moderate,  n=59 | 20.9 (6.3) | 6.2 (6.5) | 4.1 (5.3) | 70.5% | (63- 77) | 80.4% | (74- 86) |
| Severe, n=11 | 27.3 (7.1) | 11.6 (4.7) | 8.6 (4.8) | 57.5% | (47- 66) | 68.4% | (58- 78) |

^a^ Change in CCS from baseline to 3 months

^b^ Change in CCS from baseline to 12 months

Note: Percent change is calculated from baseline to 3 months and from baseline to 12 months. Confidence interval and Percent change is based on bootstrap estimation (10,000 repetitions)

**Table 4** RAND-36 scale-scores for the TBE patients at 12-months follow-up and the normal controls

| **Scales** | **TBE patients**  **n=89** | **Normative controls**  **n= 2214** | **p^a^** |
| --- | --- | --- | --- |
| **SF-36 summary scores,** mean (SD) | | | |
| PCS | 47.0 (11.0) | na | **-** |
| MCS | 50.5 (10.9) | na | **-** |
| **SF-36 norm-based scales,** mean (SD) | | | |
| Physical functioning | 81.5 (24.0) | 88.4 (17.4) | 0.008 |
| Role limitations, physical | 63.5 (43.0) | 79.7 (34 4) | <0.001 |
| Role limitations,  emotional | 78.3 (36.6) | 82.5 (31.8) | 0.282 |
| Vitality | 57.2 (24.8) | 60.3 (20.7) | 0.242 |
| Mental health | 76.7 (19.8) | 78.8 (16.4) | 0.343 |
| Social functioning | 77.1 (25.5) | 86.1 (21.8) | 0.001 |
| Bodily pain | 75.5 (26.4) | 75.7 (25.6) | 0.943 |
| General health | 67.2 (23.2) | 77.4 (21.8) | <0.001 |

^a^p for differences between the TBE patients at 12 months follow-up and the reference group

Higher score indicates greater Quality of life

Statistical significance at the level p<0.05 is shown in red text

**Table 5**: SF- 36 scores in all participants and grouped by FSS≥4 vs FSS<4 at 12-month follow-up

| **RAND-36 domaine** | **All participants**  **n=89**  Median, IQR | **FSS ≥ 4**  **n=37**  Median, IQR | **FSS < 4**  **n=52**  Median, IQR | **p^a^** |
| --- | --- | --- | --- | --- |
| Physical function | 95 (75-100) | 70 (50-95) | 100 (93-100) | <0.001 |
| Role Physical | 100 (25-100) | 25 (0-50) | 100 (100-100) | <0.001 |
| Role emotional | 100 (67-100) | 67 (0-100) | 100 (100-100) | <0.001 |
| Vitality | 60 (35-80) | 35 (20-45) | 78 (65-83) | <0.001 |
| Mental health | 84 (68-92) | 64 (52-76) | 88 (84-92) | <0.001 |
| Social function | 88 (63-100) | 50 (50-63) | 100 (88-100) | <0.001 |
| Body pain | 90 (55-100) | 45 (35-78) | 100 (90-100) | <0.001 |
| General health | 70 (50-85) | 50 (35-60) | 83 (70-95) | <0.001 |

^a^p calculated for comparison between FSS≥4 vs FSS<4

Statistical significance at the level p<0.05 is shown in red text
